# *“We’re on the journey now together”*: a qualitative examination of "close others’" perspectives on barriers and facilitators to help-seeking for eating disorders

**DOI:** 10.1186/s40337-026-01626-6

**Published:** 2026-05-14

**Authors:** Jessica Wilkins, Lucy Gallagher, Karina Allen, Ulrike Schmidt

**Affiliations:** 1 Centre for Research in Eating and Weight Disorders, Institute of Psychiatry, Psychology and Neuroscience, King’s College London, London, UK; 2 South London and Maudsley NHS Foundation Trust, London, UK

**Keywords:** Eating disorders, Close others, Family, Partners, Social networks, Help-seeking, Under-served, Caregivers, Carer

## Abstract

**Background:**

Eating disorders (EDs) are serious illnesses which profoundly impact individuals and the people close to them. Despite robust evidence that early intervention improves clinical outcomes, help-seeking by individuals with EDs is low and long periods of untreated illness are common. Social support networks can facilitate help-seeking, although most research has focused on the experiences of parents supporting adolescents with anorexia nervosa (AN) to seek help and engage in treatment. The present study sought to explore the perspectives of a broadly defined group of ‘close others’ on help-seeking for all EDs.

**Methods:**

Twelve qualitative interviews were conducted with ‘close others’ - partners, siblings, extended family members and parents – who had experience of individuals with a broad range of EDs including AN, bulimia nervosa (BN), avoidant/restrictive food intake disorder (ARFID), and other specified feeding or eating disorder (OSFED). Data were analysed using reflexive thematic analysis.

**Results:**

Themes were categorised into barriers and facilitators of help-seeking, alongside the impact on close others. Barriers identified by close others could be categorised into three main groups: close other factors (e.g., limited knowledge/ confidence in identifying the problem), patient factors (e.g., ambivalence), and systemic factors (e.g., restrictive service criteria and long wait times). Close others facilitated help-seeking by educating themselves about EDs, learning to navigate healthcare systems and actively working to reduce their loved one’s ambivalence about help-seeking. Close others reported several negative impacts on their well-being, including guilt, stress and frustration, social isolation and challenges juggling competing responsibilities.

**Conclusions:**

The findings of this study demonstrate that broadly defined social networks can play an active role in supporting individuals to seek help for EDs. These findings should be interpreted with caution given that the sample was predominantly White and largely comprised individuals with experience of AN. Interventions aimed at improving help-seeking for ED should draw on social support networks as one way of improving access to treatment. Inclusive service models, which involve close others in care, should offer both psychological and practical support to help reduce carer burden and distress.

**Supplementary Information:**

The online version contains supplementary material available at 10.1186/s40337-026-01626-6.

## Abstract (plain language summary)

Eating disorders (EDs) are serious mental illnesses that affect both the people who have them and the people close to them. Getting help early can make recovery more likely, but many people with EDs find it hard to ask for help. We interviewed partners, siblings, parents and other family members of people with different EDs. We asked them about their experience supporting their loved one to get help. From these interviews, we found several challenges and supports for getting help. Challenges included not understanding the problem, not knowing where to get help, and the person with the ED being unsure about wanting help. Close others played an active role in helping their loved one to seek help by learning more about EDs, finding out about available services, and helping with practical tasks like booking appointments. Close others described feeling stressed, frustrated and guilty. Strategies to help people get support for EDs should recognise the important role of their social networks. Education about EDs should be aimed at the general public so more people understand the problem and how to help.

**Supplementary Information:**

The online version contains supplementary material available at 10.1186/s40337-026-01626-6.

## Background

Eating disorders (EDs) are serious psychiatric illnesses associated with significant physical, psychological and psychosocial impacts [1] and have the second highest years of potential life lost among mental illnesses [2]. Early intervention is associated with improved outcomes, including shorter duration of untreated illness, reduced need for more intensive treatments [3], and improved functional outcomes [4]. Despite this, treatment-seeking for people with EDs is low, and many experience symptoms for years before accessing care [5].

Research has explored barriers and facilitators to help-seeking for EDs, to inform strategies that enable earlier treatment engagement. Barriers include individual factors (e.g. stigma, denial of the problem), systemic (e.g. service access, affordability) and demographic (e.g. gender) factors, with social support consistently identified as a facilitator of help-seeking for EDs [6–9]. However, help-seeking is a dynamic, multidimensional process shaped by a range of such factors. The candidacy framework captures this complexity, proposing seven dimensions through which individuals negotiate access to care. These dimensions include [1] identification of the problem, [2] navigating health services, [3] accessibility of services, [4] presenting to health services and seeking help, [5] decision making by health care professionals, [6] deciding whether to accept the treatment offered, and [7] structural factors that influence the process of help-seeking [10, 11] (Table 1). The framework has been used to examine access to a range of health care services for both physical health problems e.g. [12] and mental health problems e.g. [13], but has not previously been applied to help-seeking for EDs.

Research on EDs help-seeking has largely focused on families [14], particularly parents of adolescent girls with anorexia nervosa (AN) [15, 16]. These studies highlight the role that families play in identifying the problem, navigating healthcare systems, and advocating for treatment [16, 17] all of which are key candidacy dimensions. Yet perspectives from wider support networks of ‘close others’, such as friends, colleagues, and community members, remain limited. This is notable given evidence that some groups are more likely to seek support from non-family members regarding their ED [18]. For example, emerging adults are more likely to describe ambivalence about involving family members in treatment [8], people from certain ethnic minority groups have reported they are more likely to ask for help from non-family members (e.g. friends) [19, 20], and individuals with binge eating disorder (BED) and bulimia nervosa (BN) report less support from family compared to people with AN [21]. These varied close interpersonal relationships, encompassing both family and non-family members, have collectively been referred to as “close others” in ED literature [22].

Family involvement in ED treatment is also associated with improved clinical outcomes. This is well established in child and adolescent services, where family therapy is the frontline treatment [23]. Family involvement is also encouraged in early intervention models, such as the UK-based First Episode Rapid Early Intervention for EDs (FREED) programme [24, 25] and the Maudsley model of treatment for AN [26]. However, most research has focused on family involvement in the treatment of AN [27]. Given that many individuals with EDs experience multiple diagnostic presentations over time, and that clinical features frequently overlap, it is important to broaden our understanding of help-seeking processes beyond AN to encompass the full spectrum of EDs [28].

Families play a significant caregiving role for people with EDs and are profoundly impacted [29]. Parents describe negative impacts to physical and mental health, financial burdens from treatment costs, and reduced work capacity due to caring responsibilities [30–32]. Siblings also report major life disruptions and inadequate systemic support [33, 34], and partners are at higher risk of experiencing mental health problems including anxiety and depression [31]. However, there is a lack of research seeking the perspectives of a wider social network, including extended family members and friends, about help-seeking for EDs as well as the impact on their own well-being.

This study explored the experiences of a broadly defined group of ‘close others’, including extended family members and partners, who had supported someone to seek help for an ED. It aimed to advance understanding of the barriers and facilitators of help-seeking from this perspective, addressing the question: “*how do ‘close others’ experience and make sense of the process of supporting an individual with an ED to seek help?”*

**Table 1:** Candidacy framework as proposed by Dixon-Woods et al. 2006

Dimension	Summary
Identification of candidacy	Individuals recognise a problem and come to see themselves as deserving of care.
Navigation of services	Understanding where to go, how to access services, and how to move through the healthcare system.
Permeability of services	How easily services can be entered or accessed. Includes physical accessibility, appointment availability, and eligibility criteria.
Appearances at health services	Individuals’ actual presentation at services including how they communicate the nature of the problem.
Adjudications	Clinical decisions made by healthcare providers about whether someone is eligible for care.
Offers of, and resistance to, services	Offers for treatment and the individuals’ response including engagement or discontinuation of help-seeking.
Operating conditions and local context	The broader structural or organisational conditions within which services operate. Includes policies.

Dixon-Woods, M., Cavers, D., Agarwal, S., Annandale, E., Arthur, A., Harvey, J., Hsu, R., Katbamna, S., Olsen, R., Smith, L., Riley, R., & Sutton, A. J. (2006). Conducting a critical interpretive synthesis of the literature on access to healthcare by vulnerable groups. BMC Medical Research Methodology, 6, 35.

## Methods

### Design and ethics

This qualitative study used semi-structured interviews to explore the perspectives of close others of individuals with EDs. Ethical approval for the study was obtained via the King’s College London Research Ethics Committee (Reference: HR/DP-24/25-43192). All participants provided written informed consent prior to participation.

### Sample

Close others were defined as family members (including siblings), friends, and partners, reflecting evidence supporting the role of wider support networks in help-seeking [18]. Participants were recruited online via the research team’s website (edifyresearch.co.uk), an ED charity in the UK (BEAT Eating Disorders; beateatingdisorders.org.uk), an established charity supporting families of individuals with EDs (F.E.A.S.T.; feast-ed.org), and via carer support groups. Participants were also recruited through snowball sampling from a study targeting under-served groups in EDs [35], a strategy shown to be effective in engaging hard-to-reach participants [36]. Sample size was guided by previous studies of carer experiences and EDs [16], practical constraints related to fraudulent participant recruitment (detailed below), and an assessment of information power indicating that the final sample was sufficient for the analysis [37].

### Procedure

Participants first completed an online demographic questionnaire via Qualtrics (Qualtrics, Provo, UT) before taking part in a semi-structured interview. Interview questions were informed by guides used in two previous studies from our group examining help-seeking for EDs [8, 35] and invited participants to narrate their loved one's help-seeking journey, including, “What steps did your loved one take to address their eating difficulties?”, as well as their own experiences, “What has been the impact on you as a close other of someone struggling with an eating disorder?”. The full interview guide is available in the Supplementary Materials (ST1).

Interviews lasted 30–60 min and were conducted online via Microsoft Teams by researcher JW. With participant consent, interviews were audio- and video-recorded and transcribed verbatim. JW is a registered psychologist with extensive training and experience in conducting interviews; participants showing signs of distress were offered breaks and signposted to relevant support resources.

### Participant verification

During recruitment, we encountered two instances of suspected fraudulent participation. Below we outline the evidence used to reach this conclusion, consistent with emerging evidence on detecting such cases in qualitative research [38]. Several expressions of interest were received in quick succession, sharing similar email configurations and phrasing. Suspected fraudulent participants provided minimal information about eligibility, in contrast to emails received from other interested potential participants who typically included brief self-introductions. Both suspected fraudulent participants declined to enable their video during interviews, permitting only audio recording, and gave repetitive, vague responses. For example, both participants spoke exclusively about their reported close other engaging only in ‘nutritional counselling’ for their ED.

Researcher: *And then what steps did your friend take to address her eating difficulties? *

Participant: *Uh, she started going for nutritional counselling and medication.*

Researcher: *Do you know where she had that treatment or what it entailed? *

Participant: *Uhh I communicated to her and she just told me she went for a nutritional counselling and she was given some medications.*

The interviewer (JW) raised concerns about these participants, which were reviewed by the wider research team (JW, KA, US) through transcript and audio examination. Demographic data from Qualtrics revealed identical IP addresses for both suspected fraudulent participants. Thus, on balance, the evidence suggests that these participants were fraudulent: (1) Similar communication patterns in expressions of interest [39]; (2) Focus on financial renumeration [39]; (3) Repetitive, vague answers to interview questions [40]; (4) Completion of questionnaires from the same IP address [41]. Data from these participants were excluded from analyses.

### Data analysis

Data analysis followed Braun and Clarke’s (2022) phased approach to reflexive thematic analysis: data familiarisation, initial code generation, theme identification, theme review, theme definition, and report writing [42].

### Reflexive practices and researcher roles

Coding was conducted by two researchers. JW is a White, cisgender woman and a psychologist working clinically in EDs within the UK private sector. LG is a White, cisgender woman and a researcher with lived experience as a carer in physical health contexts. These clinical and personal perspectives may have shaped interpretation during coding and theme development. JW and LG met weekly to review transcripts, with reflexivity supported through note-taking and regular team discussions, including monthly meetings with the wider research team. Discrepancies were explored through discussion to deepen understanding, and to ensure that themes remained grounded in participants’ accounts. In addition, as participants were aware of JW’s clinical role, this may have influenced what was disclosed, with some potentially less likely to speak openly about negative experiences with health professionals.

### Analytical framework

Researchers conducted experiential reflexive thematic analysis guided by a critical realist approach, which conceptualises participants’ experiences as socially situated. This approach was considered most appropriate given that help-seeking and engagement with services are shaped through interactions within interpersonal relationships and healthcare contexts. Patterns in participants’ accounts of help-seeking were found to align with the dimensions of the candidacy framework. This framework was therefore used as an interpretive lens to support the presentations of findings.

### Coding and theme development

Themes were generated inductively through a reflexive analytic process grounded in participants’ accounts, rather than being predefined by the interview guide or specific topic areas. Once initial themes were identified, researchers noticed strong connections to existing help-seeking research examining experiences of carers (e.g. 9, 14—17), and the candidacy framework for understanding access to healthcare [10], and these provided additional lenses through which researchers made sense of and organised the data, in keeping with Braun & Clarke’s (2022) inductive and deductive orientations to data coding [42]. Software Atlas.ti for Mac (Version 25.0.1) was used to help with recording and organising of themes but was not used to establish coding reliability or to conduct further analysis.

### Participant characteristics

Fourteen interviews were conducted; however, two participants were excluded from analysis due to concerns regarding fraudulent participation, leaving 12 participants for analysis. Participants ranged from 20 to 60 years of age (median = 35). Four were partners (33%), three were other family members (sibling, children, extended family relations; 25%), and five were parents (42%) of an individual with an ED. Participants reported experiences with various ED diagnoses including AN, bulimia nervosa (BN), avoidant/restrictive food intake disorder (ARFID) and other specified feeding or eating disorder (OSFED). Their loved ones experienced a range of specialist ED treatment, including both inpatient and outpatient treatment across both public (NHS) and private sectors. Demographic characteristics are summarised in Table 2.

**Table 2:** Summary of participant characteristics (*N* = 12)

Age	20—60 years	Median = 35
Participant Gender – n (%)		
	Male	3 (25)
	Female	8 (67)
	Transgender	1 (8)
Relationship – n (%)		
	Partner	4 (33)
	Parent	5 (42)
	Sibling	1 (8)
	Child	1 (8)
	Aunt	1 (8)
Participant Ethnicity – n (%)		
	White	11 (92)
	Asian	1 (8)
Loved One’s Diagnosis* – n		
	AN	9
	BN	2
	ARFID	2
	OSFED	1
Loved One’s Gender – n (%)		
	Male	1 (8)
	Female	8 (67)
	Transgender	3 (25)
Loved One’s Ethnicity – n (%)		
	Asian	1 (8)
	Mixed ethnic background	1 (8)
	White	10 (83)

*Some participants reported that their loved one had received more than one ED diagnosis over their lifetime; therefore, percentages are not reported

## Results

### Thematic analysis

#### Close others’ perspectives on barriers and facilitators to help-seeking

##### Barriers

Participants described a range of barriers to help-seeking, which we interpreted within the candidacy framework as reflecting challenges across multiple dimensions of the help-seeking process, and organised into 3 main themes: their subjective experience of navigating uncertainty, patient ambivalence and marginalisation, and their experience of feeling locked out of treatment due to systemic barriers. A summary of themes is described in Table 3.

**Table 3:** Summary of themes from thematic analysis

Barriers		
	Close others navigating uncertainty	Lack of knowledge Difficulty understanding nature of the problem
	Patient ambivalence and marginalisation	About help-seeking About family involvement
	Locked out of treatment	Long waiting lists
		Inclusion/exclusion criteria
		Service commissioning
		Primary care knowledge
Facilitators		
	Positive interactions with health professionals	
	Close others’ resourcefulness mitigating barriers	
	Physical health problems	
Close others’ experiences of help-seeking		
	Negative impact of ED	On family system
		Social isolation of close other
	Navigating internal tensions and burden	Financial
		Competing responsibilities
		Time
	Parallel journey	Emotional highs and lows
		Progress is not linear
		Worry about relapse
	Need for support	Social/peer
		Individual

#### Close others navigating uncertainty

As described in the candidacy framework, the process of identifying the ED as a problem was a theme which emerged across all participant interviews. Participants described limited knowledge or confidence in recognising EDs as a barrier to help-seeking. These accounts appeared to be shaped by stigma surrounding EDs and stereotypes about the kinds of people who are presumed to be affected. For instance, some participants initially struggled to recognise their loved one’s difficulties as an ED because they had internalised the assumption that EDs occur only in girls. Many described that, in hindsight, there was a considerable gap between the onset of symptoms and the point at which they, or their loved one, identified the need for professional help. Some reported overlooking early signs of disordered eating, such as skipping meals, avoiding categories of food, and engaging in compulsive exercise. Many participants described that they did not notice incremental changes in their loved one, due to living in proximity and secrecy surrounding ED behaviours. This appeared to normalise changes, making it difficult to recognise the extent of the problem. In many accounts, it was feedback from an even wider social network that disrupted this normalisation and helped them identify the problem.

I just didn’t piece it together…that it was deeply problematic… until about a year had passed since the first day of skipping a meal, I only kind of clocked it when someone asked me the question, they said is, ‘is everything okay with [partner]? ’ (partner, AN).

#### Patient ambivalence and marginalisation

Participants described witnessing a tension in their loved ones. Although the person with the ED appeared to recognise others’ concerns, they simultaneously resisted letting go of the ED. This reflects the egosyntonic nature of EDs, in which thoughts and behaviours associated with the disorder can become linked to an individual’s values, identity and sense of control. As a result, seeking help may be experienced as risking the loss of aspects of the self that feel meaningful or protective.

She once said to me…I’ve never actually been honest with the doctor because I’ve never actually wanted to… I don’t want to let go of it (adult child, AN).

Some participants described their loved one’s ambivalence about involving them in appointments, and ultimately, in treatment. This was difficult for participants, who felt they had important information that could contribute to care. This theme was evident across parents, partners, and siblings, and reflects both structural and relational aspects of help-seeking. Within the ‘navigating services’ dimension of the candidacy framework, participants described being limited in their ability to support their loved one in presenting to services and negotiating access to treatment, which they perceived as hindering help-seeking.

having people that live with and understand that person to…work collaboratively with the service to make it more tailored. I think it’d be so beneficial (partner, ARFID).

even when I tried to have conversations and tried to explain and tried to…share that voice really wasn’t heard (sibling, AN).

Some participants’ accounts suggested that marginalised characteristics may have shaped their loved one’s experience of help-seeking across dimensions of the candidacy framework. For example, a participant whose partner with an ED was from an ethnic minority background described how cultural norms discouraged talk of mental health, impeding both their partner’s ability to recognise the problem as well as undermining their confidence in presenting to services. This suggests that help-seeking for an ED was experienced as a socially situated process and reflected participants’ perceptions of possible self-stigma, which appeared to negatively shape their loved one’s help-seeking experience.

I understand how difficult it must have been for her to grow up in that kind of environment, knowing that you can’t share…be open in that sense because…you’re not going to be taken seriously [by family]. And then you…think, oh, am I all alone in something like this? (partner, ARFID).

In parallel, gender identity appeared to shape encounters with services. Some participants described perceived mismatches between their loved one’s identity and their perception of a gendered design of available services, which contributed to feelings of exclusion. From the perspective of the candidacy framework, these accounts can be interpreted within the ‘offers of and resistance to services’ dimension, as they appeared to influence willingness to accept treatment.

lots of that behaviour was labelled aggressive…it felt to me like teenage boy behaviour, but that didn’t…sit very comfortably in a…girl-heavy eating disorder service (parent, OSFED).

#### Locked out of treatment

Participants identified several systemic barriers to help-seeking, contributing to a feeling of being locked out of treatment. They described how their loved ones were unable to access care, even when they wanted to, due to structural features of ED services. Many described limited ED knowledge among GPs, who were seen as gatekeepers to support. Within the candidacy framework, these accounts can be interpreted in relation to the ‘adjudications’ dimension, highlighting the role of clinician decision-making in shaping the help-seeking process.

I said I was concerned about her diet and he [GP] just turned around in front of her and gave me a lecture and said, ‘you know, it’s her choice… she can decide what she wants. If she wants to be a vegan.’ And I just thought, thanks…I don’t think that was really…well what we needed (parent, AN).

Participants described several factors consistent with the ‘permeability of services’ dimension of the candidacy framework. For example, difficulty accessing ED specialist treatments, with long waiting lists even after referral. These delays were understood as missed opportunities to capitalise on periods of willingness to seek help due to structural barriers.

The common theme that we’ve…run into when we’ve been trying to access eating disorder healthcare is that that he’s always…on the lower end of normal as it were… you’re underweight but you’re not concerningly underweight. So it’s always the narrative …it’s not low enough for us to give a shit, basically…it’s bad, but you’re not actively dying. And that has been like reinforced through every kind of service he’s accessed. And that’s had a big impact on him. (partner, ARFID).

Participants also described how commissioning of ED services in healthcare systems also constrained access. Individuals whose ED was not commissioned by local NHS services were often directed to seek privately funded treatment. For those unable to afford this, it represented an additional barrier and shaped perceptions of the suitability of care offered. Within the candidacy framework, this extends the ‘permeability of services’ dimension and relates to ‘offers of and resistance to services’, as the nature of the treatment offered influenced whether individuals were able to engage.

I don’t think it’s [ARFID] taken as seriously as things like anorexia… and that has been reinforced through every kind of service he’s accessed. And that’s had a big impact on him. And his own self efficacy as well… his attempts to actually access healthcare for eating disorders, it’s just dwindled so much because he’s always got into his head…I’m not bad enough…I’m not unwell enough to basically demand access to this (partner, AFRID).

### Facilitators

#### Positive experience with health care professionals

Within the dimensions of ‘presenting to services’ and ‘adjudications’ of the candidacy framework, participants identified several factors that promoted help-seeking and engagement with treatment. Positive, validating interactions with healthcare professionals reduced shame and stigma, reinforcing that help-seeking was appropriate and worthwhile.

I think me being there, the doctor…responding well to what I was talking about and how she was asking questions, I think… [my sister] was like…maybe I could accept this help in this format (sibling, AN).

But she [health care professional] did help…I think just for her to hear someone say… ‘we need you to be in a good place, your brain needs to be fully functioning for you to make the right decisions. You can’t make decisions when you’re undernourished.’ To hear somebody …say that to her, it made her a little bit more determined (parent, BN).

#### Close others resourcefulness mitigating barriers

Although most participants had little knowledge of EDs prior to their close other’s illness, all reported mobilising carer agency once the problem was recognised. They positioned themselves as active learners, engaging with psychoeducational resources through online web searches and navigating available care pathways via third sector organisations and carer support groups. Increasing their knowledge and understanding of EDs facilitated help-seeking, and directly mitigated barriers, by enabling them to better understand the challenges faced by their close other, navigate healthcare systems to access specialist treatment, and acquire skills to support their loved one in daily life.

in the beginning…I thought it’s very easy, just eat more. But talking to them [support services]…helped me really understand, the way I can help her (partner, ARFID).

In describing how they mobilised available resources and persisted in seeking appropriate support, close others appeared to engage with dimensions of the candidacy framework in parallel with their loved one. They described a dynamic process of negotiating access to care, even when this created conflict, which participants interpreted as reflecting their loved one’s readiness rather than an absence of need for help.

She was just angry. Really angry… because I took her back, you know, I made another appointment and I got her in six weeks later…and she did go, so somewhere she knew she needed the help (parent, AN).

Later, close others used their increasing knowledge about EDs and practical skills to challenge their loved one’s ED directly, supporting further help-seeking and ongoing treatment. In doing so, they were actively addressing patient ambivalence as a barrier to help-seeking. In these accounts, participants appeared to draw on their relational knowledge and used problem-solving to enhance their loved one’s readiness for change.

Knowledge is definitely power… If you kind of just try and muddle through based on just how you’re both feeling, you’ll probably never really get there…because I certainly wouldn’t have done if I hadn’t have read into it more… It’s not logical, someone is hungry, they should eat… but I just think you need to understand the emotional side of it (partner, AN).

#### Physical health problems facilitate help-seeking

Some participants reported that seeking help for physical health problems facilitated their loved one to seek help for EDs by offering an opportunity to present to services. This may be interpreted as a pattern shaped by the stigmatisation of mental health difficulties, whereby seeking help for physical health problems was perceived by participants to be more acceptable to their loved ones.

[the GP] told her that she had got osteoporosis. That was a real wake up…she turned around to me and said what have I done to myself? It made her realise that actually this was really serious (parent, AN).

### Close other experiences of supporting someone to seek help

#### Negative impact of the ED

Participants spoke about a range of negative impacts on themselves as close others, along with wider family networks. All participants described feeling isolated as a close other supporting someone with an ED. Experiences within their broader social support networks ranged from well-meaning but ultimately unhelpful advice to judgement and dismissal of their experiences.

the biggest challenge when it comes to discussing things like this with other people… I think that you have to have some lived experience to…truly understand what it really is like…friends always want to be supportive, but like it’s like… ‘can you be bothered to deal with that?’ I find that to be really challenging…because people … don’t understand… how difficult it is. (partner, ARFID).

Supporting someone with an ED had a negative impact on their ability to maintain their own social connections which further contributed to isolation and loneliness. The emotional labour of caring for someone with an ED was described in participants’ accounts as largely invisible to others, with this invisibility understood as contributing to experiences of isolation, loneliness, and feeling misunderstood.

I’ve missed things because I’ve been with her. And yeah, people don’t get it… and you try and explain how it is, but they still don’t really get it. And then sometimes you find yourself thinking, oh, I’m just, I’m just not going to bring it up tonight. I’ll just try and… then they probably think, oh, she’s not talking about it (parent, AN).

#### Navigating internal tensions and burden

Participants described the challenges of navigating internal tensions, juggling multiple, and often competing, responsibilities while supporting someone with an ED to seek help. This included managing responsibilities related to employment, but also relationships with other family members, including caring for aging parents.

I feel huge guilt that I work long hours and that I should be at home to be supportive of her and have contemplated whether or not I need to step back from work a bit or even stop entirely, and I might even consider it. Except we can’t afford for me not to (partner, ARFID).

There was also a significant impact on daily life, with participants describing how mealtimes, food choices, time spent preparing food, and family celebrations were impacted in the context of the ED. Burden emerged in the analysis as a repeated interpretive thread, with participants describing the pervasive impact of the ED on their daily functioning and relational interactions.

small things like…wanting to get together with other family members for their birthdays…having to think about what time we can go or how I can make sure she’s eaten before we go so that she didn’t have to eat in a social setting… all of those things, Christmas, birthdays, all of those events (partner , AN).

Many participants described the financial burden of supporting their loved one, either by privately funding treatment or because of the necessity of working reduced hours to attend multiple appointments.

And that makes me that makes me cross and exhausted…I’ve had to change my work. So I now work weekends, so I’m able to…. transfer care to my husband at the weekend. During the week I live the arrangements… how long is that going to be in my life? (parent, OSFED).

Others expressed guilt and frustration that they could not afford the treatment they perceived their loved one needed but could not access through public health services. This highlighted how access constraints shaped close others’ own understandings about illness and their described sense of obligation to ensure their loved one received treatment.

We’re not wealthy people, we don’t have… private healthcare. But you do what you have to for your kids…the doctor said the waiting list for [NHS service] is probably about eight months and recommended that we kept our appointment with…the private eating disorder specialist. So we did (parent, AN).

we need some sort of family therapy as well…including her brothers…but we just can’t afford to do it whilst we’re doing all that (parent, AN).

#### Parallel journey (navigating hope and worry)

All participants described a parallel journey alongside their close other, involving both emotional and practical dimensions. This journey seemed to be instinctive, with participants describing a sense of obligation that drew them into sustained emotional and practical labour. Participants understood their journey as being intimately entwined with the experience of their loved one, characterised by shared learning about EDs.

[partner]’s the one going through it. But it is her whole family, that are…impacted and we’re on the journey together. We’re all going through this with you. It’s not just you. (partner, AN).

Participants experienced several negative feelings in the context of supporting their close other to seek help for an ED including fear, helplessness/hopelessness, guilt, anger, frustration, and loss. Participants described experiencing, at times, overwhelming worry about their close others’ well-being and this was constructed by participants to be distinct to the emotional journey they perceived their loved one to be experiencing in the context of help-seeking for their ED. Participants’ accounts suggested that the stress of navigating healthcare systems unfolded alongside the emotional burden of witnessing their loved one’s suffering.

I think as a carer that impact is, is so huge… the worry of it never getting better….being so acutely aware that I could still lose him. And that that’s a that’s a very real thing I live with as his carer (parent, OSFED).

it’s difficult sometimes to know…how to approach some of her behaviours in a supportive way. It’s immensely frustrating to watch somebody that you love, making choices for themselves that, that aren’t…their, you know, best interests (partner, AN).

All participants described persistent worry about the risk of their close other relapsing or decompensating. This concern appeared to permeate their daily lives, shaping how they monitored and interpreted even subtle changes. They were highly attuned to changes in behaviour in an effort to prevent relapse.

It makes me feel quite… quite desperate for how life is. I worry that you know, when will I ever feel peace? I’m not sure I will, I’m not sure I will ever feel peace again. That’s something that’s quite unique to this level of mental illness (parent, OSFED).

Participants wove feelings such as pride and hope into their accounts of providing care. They described relief when their loved one accepted treatment, framing treatment as easing the burden of responsibility they had been carrying for the day-to-day care of their loved one. Participants conveyed how treatment redistributed some tasks such as facilitating meals and monitoring physical health, which previously had been the responsibility of carers. Participants positioned themselves as their loved ones’ champions, able to notice signs of improvement, recovery and engagement, even if their loved ones were finding this difficult. This experience of their loved one’s illness seemed to reduce their burden or burnout while bolstering their resilience to continue supporting them in the iterative process of help-seeking.

And also this process of, going through treatment, it has helped our bond to become strong (partner, AFRID).

if you think about her journey, you know it’s not we’ve not gone backwards, we’re moving forward. But you know, if you just looked at it sort of from a naked eye perspective, you would say everything’s worse (partner, AN).

#### Need for support

All participants recognised the value of social support for themselves as carers and spoke of the benefit of accessing both formal and informal support. Experiences accessing carer support groups were unanimously described as positive. Sessions with peers appeared validating, reduced feelings of isolation and provided practical information which enhanced their ability to support their loved one’s treatment.

the content… that was still great…but it’s the people…and you think, if… these people can do it, I can do it. You know…we’ll be okay (sibling, AN).

Social support also helped close others recognise their own need for support, including both physical and mental health support. This pattern reflects a parallel process in which recognising the demands of caregiving prompted close others to undertake their own help-seeking, often encountering similar challenges to those faced by their loved ones, including managing their own ambivalence about asking for help and navigating complex services.

something that that you learn during the carer session, is that I think you can only be a good carer if you care for yourself (partner, ARFID).

## Discussion

The results of this study confirm and extend existing findings that close others play a vital role in facilitating help-seeking for EDs [6, 9]. Consistent with prior research in parents of people experiencing AN, close others in this study helped individuals with EDs to access support by identifying the problem, addressing their close others’ ambivalence [9, 43], navigating healthcare systems to obtain specialist ED treatment, and coordinating appointments [17, 44]. In our study we found that this was the experience across a range of close other relationships (i.e. partners, adult children, siblings) and in non-AN diagnoses. This consistency across a broader range of relationships, diagnoses, and participant characteristics strengthens confidence in previous findings, suggesting that barriers and facilitators to help-seeking extend beyond the contexts in which they have previously been studied. The results of this study were interpreted through the lens of the candidacy framework, which conceptualises help-seeking as a dynamic process through which individuals negotiate access to healthcare [10]. Participants described an ongoing, iterative development of advocacy and agency, evidenced not only for individuals with EDs, but also for close others, who were involved in recognising the problem, navigating services, and negotiating with health professionals to access specialist treatment.

Previous research shows that difficulties identifying the problem and the egosyntonic nature of EDs impedes help-seeking [8, 45]. Similarly, participants in this study described their loved ones’ ambivalence and made significant efforts to encourage them to seek help. In the candidacy framework, this is the first component of help-seeking, wherein individuals and their families need to recognise they are experiencing a problem that they need help with. In their accounts, participants interpreted their loved one’s uncertainty about whether they needed help as a feature of the illness itself, rather than as an indication that their loved one did not need help. In this way, participants were ultimately able to draw on their own skills and resources to support their loved one’s engagement in help-seeking. Research in psychosis similarly indicates that family involvement supports help-seeking, particularly where ‘lack of insight’ prevents individuals recognising their need for care [46]. This suggests that involvement of close others may be especially useful in facilitating help-seeking when individuals struggle to recognise their ED as a problem.

Consistent with existing research, including the candidacy framework, participants reported that help-seeking for physical problems facilitated help-seeking for EDs [6, 47, 48]. However, close others perceived significant barriers related to presenting to services and clinical decision-making. In particular, they perceived limited ED knowledge among primary care providers [49], noting that concerns were often dismissed or redirected toward physical symptoms [14], which they saw as hindering recognition and access to appropriate care. These findings reinforce calls for improved ED awareness and screening within primary care [50, 51].

Across different relationship types, including siblings, partners and adult children, experiences supporting help-seeking were broadly consistent. This extends previous work which has mainly focused on the role of parents of adolescents, both within the field of EDs and wider mental health help-seeking literature [14, 44, 52]. Importantly, there is evidence that some individuals (e.g. individuals from ethnic minority backgrounds) may actively prefer support from friends or non-family support networks, underlining the value of broadening the focus beyond parents alone [19]. These findings may also be relevant for emerging adulthood (ages 15–25 years), a developmental period that coincides with peak ED onset, yet is marked by lower rates of help-seeking [53]. Characterised by increasing independence from parents and greater reliance on friendships and romantic relationships, emerging adults may feel ambivalent about family involvement in ED care [54]. Thus, understanding how wider social networks support help-seeking may help address unmet treatment needs and inform adaptations to existing interventions or the development of new approaches to improve access for under-served populations.

These findings also indicate that the caregiving burden of ED extends beyond parents and cohabiting relatives, with close others across other relationships providing time-intensive support. They experience similar negative impacts to those previously reported by parents and siblings, including increased risk for poor mental health and burnout alongside worry, guilt and frustration [9, 32, 34].

### Clinical implications

Several clinical implications can be drawn from this study. First, broad public awareness campaigns may reduce barriers related to poor recognition of ED symptoms and uncertainty about when and how to seek care [55]. This could help to address difficulties with navigating healthcare systems, which participants described as a major challenge [56]. Second, targeted psychoeducation for close others may equip them with strategies to reduce ambivalence. One such method might be online mobile-based interventions, such as the UK-based FREED-M, which targets readiness to change [57], or an adaptation of existing interventions designed for families of individuals already accessing treatment [27, 58].

The findings of this study demonstrate that EDs are not only individual experiences but are embedded within and impact broader social networks. However, ED services for children and adolescents conceptualise the role of family and friends differently to adult services. For children and adolescents, family-based interventions are frontline treatments and families and carers are seen as an integral part of care. In contrast, adult ED services take a more individual-based therapeutic approach with interventions such as CBT-ED primarily focused on the individual [59]. The Maudsley Collaborative Care integrates carers as part of treatment and defines them broadly as any suitable source of support [26, 60]. Similarly, early intervention programs targeting emerging adults that include family and friends demonstrate good clinical outcomes [25]. Together, these findings suggest that involving close others in treatment is associated with improved clinical outcomes. However, current guidance on family involvement remains focussed on parents, overlooking the potential contributions of non-parent caregivers, including siblings and partners [33]. Extended social networks play a significant role in supporting help-seeking and treatment as agents of change and their experiences and perspectives should therefore be included in guidance on family involvement.

Beyond improving help-seeking, our findings underscore the need for robust support for close others of individuals with EDs. Participants in this study described profound psychosocial impacts resulting from assisting their loved one to seek help for an ED. They also identified a positive impact of social support and psychoeducation, aligning with existing research demonstrating that such resources can mitigate caregiver burden and distress [61].

### Implications for policy and practice

The accounts of participants in this study offer evidence for several policy and practice recommendations which could improve care for people with EDs as well as the experiences of their close others. Systemic challenges are well documented in ED research and are identified as barriers to help-seeking across other mental health problems [52]. Evidence demonstrates that service enhancements to streamline referral processes, reduce barriers around inclusion criteria, and allowing direct referrals improves engagement with mental health treatment [62]. Eliminating BMI thresholds for services, allowing direct referral, and increasing commissioning across all ED diagnoses to bring them in line with clinical guidelines [63] could similarly improve help-seeking for EDs. Furthermore, given the financial burdens experienced by close others in this study, both in terms of paying for treatment and time off from work to provide support or attend appointments, there is evidence for the utility of financial support for carers of people with EDs. And finally, results of this study demonstrate the valuable role of both formal and informal support to carers which suggests that support for close others should extend beyond psychoeducational materials and support groups to developing social networks that carers can access over time [64].

### Strengths and limitations

A strength of this study is the range of familial relationships represented, contributing insight into how different close others facilitate help-seeking for EDs. This extends existing research, which has largely focused on the parents of White, female individuals with AN. Participants also described experiences of help-seeking for a range of ED diagnoses, including ARFID, BN and OSFED, alongside variation in gender and ethnicity of their loved ones with EDs, offering important context. We were also able to manage inauthentic participation. There has been an increase in studies reporting fraudulent participants in both online survey [41] and qualitative research studies [65], particularly in studies using snowball sampling or offering reimbursement [40].

Despite efforts to engage under-served groups (e.g. individuals from marginalised socioeconomic, ethnic, sexual/gender groups), most participants in this study were White, and many had the resources to access private treatment. Following NIHR INCLUDE guidance on involving under-served populations in research [66], recruitment strategies included collaboration with third-sector organisations serving relevant communities, participant remuneration, and flexible participation formats (e.g., online or written). However, diversity remained limited, highlighting persistent challenges in engaging carers who may be time-poor or less connected to support services [32].

Cultural norms emphasising self-reliance, alongside self-stigma surrounding mental health may further limit help-seeking among under-served groups [19, 67, 68]. Such norms may influence both awareness of available services and perceptions of their usefulness [69], and may similarly affect close others, contributing to their under-representation in research and limited engagement with support services. As White, cis-gender researchers, whose identities align with groups most typically represented in dominant descriptions about EDs, we occupy a position of relative power within the research encounter. Our position as ‘outsider’ researchers in relation to under-served communities may also have influenced those who chose to participate and what was disclosed during interviews. In future research, co-production of culturally responsive materials and research study protocols may enhance engagement by ensuring that programmes reflect the experiences and needs of diverse close others [69, 70].

Participants described investing considerable time, and in some cases, money into learning about EDs and navigating healthcare systems. This raises important questions about the experiences of individuals with fewer educational resources, language-related barriers (e.g. communicating in a non-primary language), or greater time constraints. As such, the findings largely reflect the perspectives of close others with comparatively greater access to resources, suggesting a degree of self-selection bias. This, combined with the interpretative nature of reflexive thematic analysis, limits the generalisability of our findings. Future research should prioritise engaging close others from under-served ED populations to better understand the unique experiences and perspectives of these under-represented groups.

## Conclusion

A network of close others actively facilitates help-seeking for people with EDs. Close others facilitate help-seeking by educating themselves about EDs, learning to navigate health systems and coordinating formal help-seeking behaviours. Close others experience a negative impact of EDs on quality of life; however, those who engage in social support report benefits to their well-being as well as their ability to support their loved ones. Interventions to improve help-seeking for EDs should recognise the importance of extended social support networks in fostering help-seeking behaviours.

## Electronic Supplementary Material

Below is the link to the electronic supplementary material.

Supplementary Material 1.

## Data Availability

The dataset collected and analysed during the current study is not publicly available as this may violate participant privacy. The corresponding author can be contacted with questions regarding the data set.

## Abbreviations

AN: Anorexia Nervosa
ARFID: Avoidant/restrictive food intake disorder
BED: Binge eating disorder
BN: Bulimia Nervosa
BMI: Body Mass Index
ED: Eating Disorder
FREED: First Episode Rapid Early Intervention for Eating Disorders
NHS: National Health Service
NIHR: National Institute for Health and Care Research
OSFED: Other specified feeding or eating disorder
