# Supplementary material for: “We’re on the journey now together”: a qualitative examination of "close others’" perspectives on barriers and facilitators to help-seeking for eating disorders
Source: J Eat Disord. 2026 May 14;14:157. doi: 10.1186/s40337-026-01626-6 (PMC13352777; doi:10.1186/s40337-026-01626-6)
Supplement: Supplementary file 1 — Supplementary Material 1. [file 40337_2026_1626_MOESM1_ESM.docx]

**Supplementary Materials (ST1) - Semi-Structured interview questions**

1. Can you tell me about when your loved one’s behaviours or thoughts around food/body image started to become a concern for you?

**Probes:**

- Changes noticed
- Feelings towards / understanding of changes
- Life context
- Did you speak to them about it. If yes, what happened when you did?

2.How did things progress?

**Probes:**

- Progression of symptoms
- Life context
- Their own thoughts/feelings as their loved one’s eating problems progressed over time

3.What steps did your loved one take to address their eating difficulties?

**Probes:**

- Professional help seeking (e.g. health professionals, GP, specialist treatment)
- Informal help-seeking (e.g. social support, friends)
- Information seeking (e.g. internet searches, reading books)
- When?
- Did you attend any health appointments with them?
- What was the outcome?

4.Were there any things that helped you understand what your loved one was experiencing?

**Probes:**

- Information seeking (e.g. internet searches, reading books)
- Informal help-seeking (e.g. social support, friends)
- Professional help-seeking (e.g. health professionals)

5.Did you access any support for yourself around your loved one’s eating problems?

6.Did you have any knowledge of eating disorders and who they affect?

**Probes**

- How well do they match with own identities / characteristics?
- Impact on symptoms / help-seeking

7. What has been the impact on you as a close other of someone struggling with an eating disorder?
